# Regulatory Modules Involved in the Degradation and Modification of Host Cell Walls During *Cuscuta campestris* Invasion

**DOI:** 10.3389/fpls.2022.904313

**Published:** 2022-07-06

**Authors:** Ryusuke Yokoyama, Toshiya Yokoyama, Takeshi Kuroha, Jihwan Park, Koh Aoki, Kazuhiko Nishitani

**Affiliations:** ^1^Graduate School of Life Sciences, Tohoku University, Sendai, Japan; ^2^Faculty of Science, Kanagawa University, Hiratsuka, Japan; ^3^Division of Crop Genome Editing Research, Institute of Agrobiological Science, National Agriculture and Food Research Organization, Tsukuba, Japan; ^4^Graduate School of Life and Environmental Sciences, Osaka Prefecture University, Sakai, Japan

**Keywords:** parasitic, haustorium, cell wall, ERF, regulatory module

## Abstract

Haustoria of parasitic plants have evolved sophisticated traits to successfully infect host plants. The degradation and modification of host cell walls enable the haustorium to effectively invade host tissues. This study focused on two *APETALA2/ETHYLENE RESPONSE FACTOR* (*ERF*) genes and a set of the cell wall enzyme genes principally expressed during the haustorial invasion of *Cuscuta campestris* Yuncker. The orthogroups of the TF and cell wall enzyme genes have been implicated in the cell wall degradation and modification activities in the abscission of tomatoes, which are currently the phylogenetically closest non-parasitic model species of *Cuscuta* species. Although haustoria are generally thought to originate from root tissues, our results suggest that haustoria have further optimized invasion potential by recruiting regulatory modules from other biological processes.

## Introduction

*Cuscuta* species are one of the most widespread groups of parasitic plants that severely damage economically important crops, reducing yields (Westwood et al., 2010). Parasitic plants absorb resources from the host through an invasive organ called the haustorium (Dawson et al., 1994). Haustorium penetrates the host tissue and develops into search hyphae, which elongate within the host tissue. Search hyphae differentiate into vascular hyphae upon reaching the host vasculature, followed by the establishment of a vascular connection between the parasite and the host plant (Kaga et al., 2020; Park et al., 2022). A series of haustorium development processes are involved in a variety of molecular interactions between host plants and parasitic plants (Hegenauer et al., 2016, 2020). Thus, understanding the molecular basis of haustorium development is not only important from a crop production viewpoint but also of interest from a biological perspective (Shimizu and Aoki, 2019).

Recently, molecular research on *Cuscuta campestris* provided an invaluable basis for enhancing our knowledge of haustorium development and function. For example, Jhu et al. (2021) demonstrated that CcLBD25 is a crucial regulator of *C. campestris* haustorium development including haustorium initiation. Furthermore, three genes (*CcHB7, CcPMEI*, and *CcERF1*) were recently identified as putative key regulators of *C. campestris* haustorium organogenesis (Jhu et al., 2022). Previously, we also observed that *C. campestris* utilizes host-produced ethylene for the proper growth of search hyphae, and penetration into the host tissue is facilitated by the degradation and modification of host cell walls (Narukawa et al., 2021; Yokoyama et al., 2021). Additionally, we performed transcriptomic analysis using an *in vitro* induction system to characterize xylem vessel cell differentiation in the haustorium of *C. campestris* (Kaga et al., 2020). Several aspects of the haustorium have been evaluated, but the molecular-level understanding of haustorium development, especially regarding transcriptional regulation, remains incomplete.

To understand the transcriptional regulation of haustorium development, we comprehensively characterized the TFs involved in haustorium development. This study focused on two *APETALA2/ETHYLENE RESPONSE FACTOR* (*ERF*) genes that displayed a significant expression correlation with the cell wall enzyme genes during the haustorial invasion. The *C. campestris* ERFs are orthologous to tomato SlERF52, which regulates genes encoding abscission-associated cell wall enzymes (Nakano et al., 2014). In plants, abscission is generally defined as the process that detaches leaves or flower organs from the remainder of the plant body. In this process, cell wall degradation is facilitated by the activation of cell wall enzymatic genes, which are orthologous to the *C. campestris* cell wall enzyme genes involved in haustorial invasion (Yokoyama et al., 2021). Together, the *C. campestris* ERFs might activate the transcription of the cell wall enzymatic genes in haustorial invasion.

## Materials and Methods

### Comparative Analysis of Gene Expression Profiles in Haustoria of *Cuscuta campestris* Parasitizing *Nicotiana tabacum* Stems and Non-living Materials

RNA-seq analysis was performed by using *C. campestris* stem coiling around rods made of non-living material (dried bamboo rod) or living material (stem of wild type *Nicotiana tabacum*). Seeds of *C. campestris* were germinated, grown for 7 days, and coiling was induced, as described previously (Hozumi et al., 2017). The time at which *C. campestris* coiled around the host *N. tabacum* stem was designated 0 h after coiling (hac). Samples of coiling stems of *C. campestris* were harvested at 0, 24, 48 hac, and total RNA was prepared as described previously (Shimizu et al., 2018). Sequencing libraries were constructed by using TruSeq RNA Library Preparation Kit v2, and read by using Illumina HiSeq 2500 platform (Illumina Inc., San Diego, CA, United States.) to obtain 100 nt-long single-end reads. Quality and read length distribution of the raw data in the FASTQ format were checked using FastQC.^1^ Barcode removal and adapter sequence trimming was performed with the Trimmomatic (Bolger et al., 2014), followed by quality control (-q 20 -p 80). Mapping to *C. campestris* transcripts (Vogel et al., 2018) was performed by using HISAT2 and Stringtie (Pertea et al., 2015). Gene Ontology term enrichment analysis was performed by using web tool provided by The Arabidopsis Information Resource (TAIR).^2^ Run data are registered in DDBJ Sequence Read Archive under accession numbers of DRR353312-DRR353329.

### Cluster Analysis of Gene Expression Data in Haustorium Development in *Cuscuta campestris* Parasitizing *Arabidopsis thaliana*

We performed soft clustering analysis on gene sets defined as DEGs using Mfuzz (Futschik and Carlisle, 2005) based on TPM with the RNA-seq data generated previously by our group (Kaga et al., 2020). RNA-seq data were obtained from the DNA Data Bank of Japan (DDBJ) Sequence Read Archive.^3^ A brief description of each sample is as follows. The *C. campestris* stems were harvested at 24 h after exposure to blue light and attachment to host. We designated “time 0 h after coiling” as the time point at which *C. campestris* completed coiling around the host (Hozumi et al., 2017). This stage probably corresponds to a pre-infective stage because expression of marker genes for an early infective “swelling stage” is not detected (Bawin et al., 2022). Additionally, the marker gene (e.g., Cc008373) for the early infective stage was specifically expressed at 12 hac. The exact timing when Cuscuta stem stops coiling movement around the host was determined based on the images captured by time lapse camera at 5-min intervals during the coiling and subsequent period. Tissue samples obtained at 0 hac consisted of the epidermis and cortex of the *C. campestris* at the contact site of the *Arabidopsis thaliana* (L.) Heynh inflorescence stem. Tissue samples obtained at 12, 42, and 54 hac were all derived from haustoria in the coiling regions of *C. campestris* lateral shoots parasitizing the *A. thaliana* stems. Tissue samples (*n* = 50) were transversely sectioned (100 μm) along the host stem axis, using a vibratome. Haustorial regions were excised and pooled from the transverse sections by LMD using the PALM MicroBeam (Carl Zeiss Microscopy GmbH). Total RNAs were isolated from 50 haustorial regions. Three biological replicates were prepared for each stage of the development. Tissue samples containing epidermal and cortical cells of *C. campestris* without contact with the *A. thaliana* stem were used as negative controls (-). The “flower” and “seedlings” samples were derived from *C. campestris* flower bud clusters and 7-day-old seedlings, respectively.

### Identification of Transcription Factors in *Cuscuta campestris*

A protein sequence dataset for *C. campestris* was downloaded from plaBiPD^4^ (Vogel et al., 2018). The protein sequences for each TF family of *Solanum lycopersicum* L. were obtained from PlantTFDB.^5^ BLASTP (e ≤ 1e−5) was performed to search for homologous proteins against the *C. campestris* protein sequence dataset using the protein sequences of TFs of *S. lycopersicum* as queries.

### Phylogenetic Analysis and Characterization of *Cuscuta campestris* Ethylene Response Factors

Phylogenetic analysis was performed using the *C. campestris* and *S. lycopersicum* ERF protein sequences (Zhang et al., 2022). The amino acid sequences were aligned using the Gonnet matrix in Clustal 2.1. A phylogenetic tree was constructed using the iTOL online tool.^6^ Conserved motifs of 10 ERF members of the subclade, including SlERF52, and consensus logos for the AP2/ERF domains were obtained using the software tool MEME.^7^

### Identification of *Cuscuta campestris* Orthologs of Tomato Abscission-Associated Transcription Factors

*Cuscuta campestris* orthologs of tomato abscission-associated TFs were identified based on a previous study (Ito and Nakano, 2015). BLASTP (e ≤ 1e−5) was performed to search for homologous proteins against the *C. campestris* protein sequence dataset, using the sequence data retrieved from the reference as queries. A graphical representation of the pairwise correlation between the haustorial expression patterns was constructed using the R package Corrplot.

## Results

### Identification and Classification of Transcription Factor Genes in *Cuscuta campestris*

To comprehensively identify the TF genes in *C. campestris*, BLAST searches of the *C. campestris* database were performed using the protein sequences of the tomato TFs as queries. Tomato (*S. lycopersicum* L.) is currently the phylogenetically closest non-parasitic model species of the *Cuscuta* species and serves as a model for the family Solanaceae (Kimura and Sinha, 2008). We obtained tomato TF datasets from PlantTFDB,(see text footnote 5) a useful resource for the sequence and classification of plant TFs (Tian et al., 2019). A total of 1,283 *C. campestris* genes were identified as potential TFs and then classified into 55 TF families (Supplementary Table 1).

### Transcriptome Analysis of Transcription Factors Involved in the Interaction With the Host Plant *Nicotiana tabacum*

**FIGURE 1:**
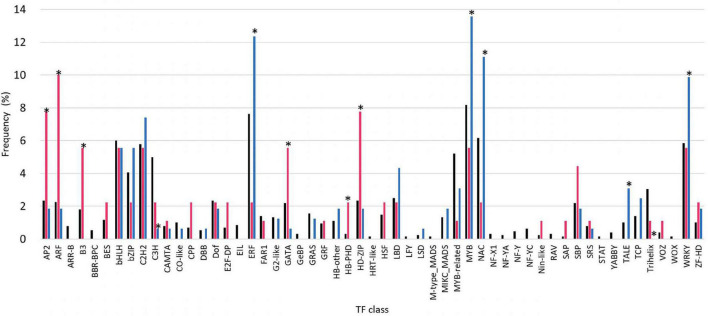
Comparison of transcription factor (TF) expression in haustoria of *Cuscuta campestris* parasitizing *Nicotiana tabacum* stems and non-living materials. TF families were classified according to the identification and characterization of PlantTFDB (http://planttfdb.gao-lab.org). Black bars indicate the population of family genes in all the TF members of *C. campestris*. Red bars indicate the population of members that showed higher expression levels in *C. campestris* parasitizing *N. tabacum* stems than rods made of non-living material (dried bamboo sticks) at 24 and 48 hac, and progressively increased of expression levels from 0 to 48 hac (fold change (FC) > 2, comparing 0 hac vs. 48 hac), in each TF family. Blue bars indicate the population of members that showed lower expression levels in *C. campestris* parasitizing *N. tabacum* stems than bamboo sticks at 24 and 48 hac, and progressively decreased of expression levels from 0 to 48 hac [fold change (FC) < 0.5, comparing 0 hac vs. 48 hac], in each TF family. Asterisks indicate significant difference at 5% level of probability among members of each TF family.

To examine the contribution of TFs to haustorium development involved in the interaction with the host, we performed the RNA-seq analysis to compare the expression levels of TFs of *C. campestris* stems coiling *N. tabacum* stems and a rod made of non-living materials. The results showed that their expression was influenced by the presence of host plant, although several types of the TF families had only the members with unchanging expression profiles (Figure 1). Among 1,283 TFs, 96 TFs showed significantly higher level of induction by the stem of living *N. tabacum* than non-living rod, while 180 TFs showed significantly lower level of induction. GO enrichment analysis demonstrated that GO terms such as regulation of cell differentiation (GO:0045595) and phyllome development (GO:0048827) were enriched uniquely among the TF genes which showed higher induction by living *N. tabacum*, and that defense response to fungus (GO:0050832), response to wounding (GO:0009611) and regulation of secondary cell wall biogenesis (GO:2000652) were enriched exclusively among the TF genes which showed lower induction by living *N. tabacum* (Supplementary Table 2).

### Identification of Transcription Factor Genes Involved in Haustorium Development in *Cuscuta campestris* Parasitizing *Arabidopsis thaliana*

**FIGURE 2:**
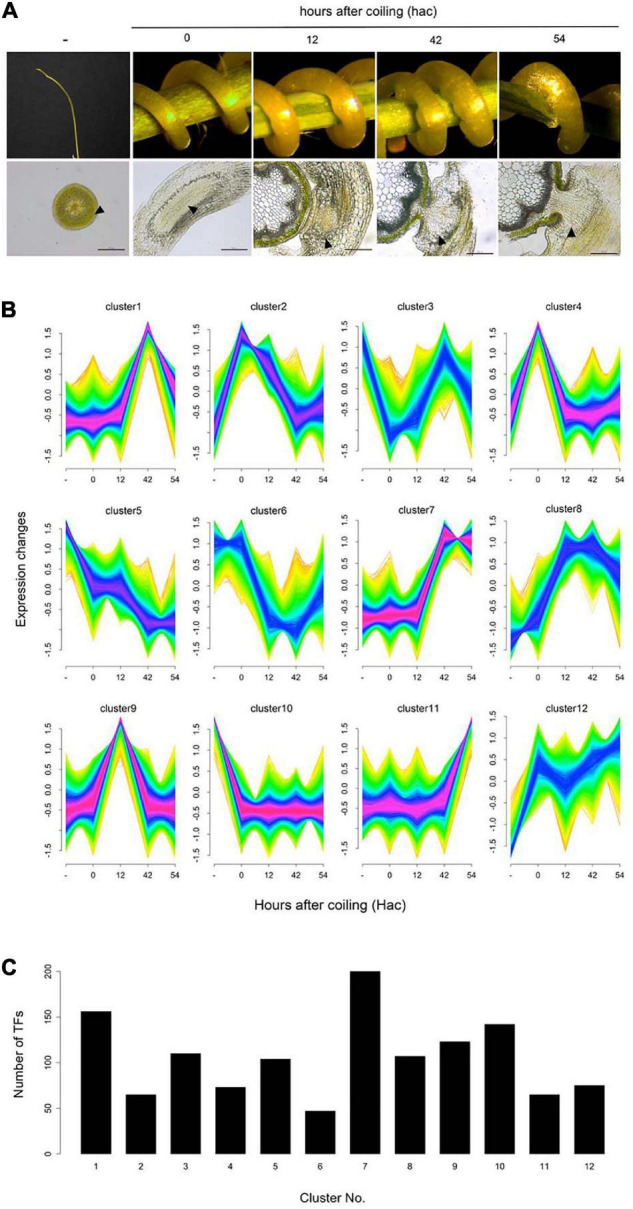
Cluster analysis of differentially expressed genes during the haustorial invasion. **(A)** Images of the samples and the sections in each stage of haustorium development. Arrowheads indicate the tissue areas collected by laser microdissection. Scale bars: 200 μm. **(B)** Clustering analysis of differentially expressed genes (false discovery rate < 0.01) using Mfuzz. **(C)** Number of the transcription factors in each cluster.

We investigated differentially expressed TF genes among different stages of haustorium development in *C. campestris* parasitizing the host plant *A. thaliana*. Using our previous comprehensive RNA-seq data for *C. campestris* haustorium development in combination with LMD (Kaga et al., 2020), clustering analysis was performed, and differentially expressed TF genes were identified in each cluster (Figure 2; Supplementary Table 3). The TF genes expressed in haustoria of *C. campestris* parasitizing *A. thaliana* coincided with those in *C. campestris* parasitizing *N. tabacum*, with some exceptions. The present results, associated with those of our previous reports, confirmed that TFs involved in vascular differentiation were predominantly expressed during the later stages of haustorium development (Supplementary Table 4; Kaga et al., 2020). We further characterized the TF genes predominantly expressed at the initial invasion stage (clusters 2 and 9 in Figure 2B), during which haustoria penetrated the host tissue by specific biochemical degradation and modification of host cell walls. The members of clusters 2 and 9 contained orthologs to Arabidopsis TFs involved in stress responses and developmental processes (Supplementary Table 5). However, we focused on *C. campestris* orthologous *SlERF52* genes because SlERF52 is known to regulate cell wall enzymatic activities in tomatoes (Nakano et al., 2014).

### Characterization of Orthologous SlERF52 in *Cuscuta campestris*

**FIGURE 3:**
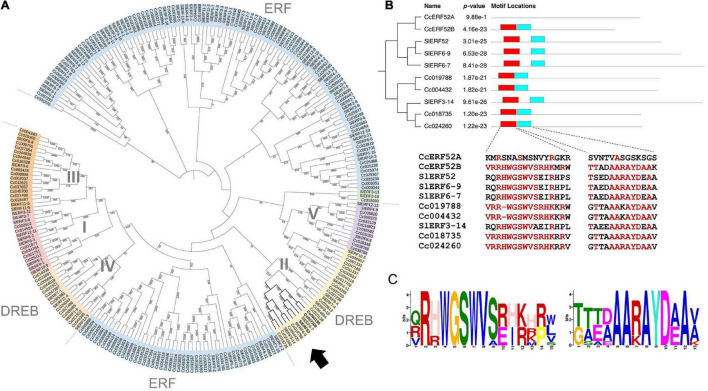
Phylogenetic analysis and conserved protein domain of the *C. campestris* SlERF52 orthologous genes. **(A)** Phylogenetic relationships of the ERF proteins from *C. campestris* and tomato. The phylogenetic tree was constructed by iTOL (https://itol.embl.de). Arrow indicates the subclade, which includes SlERF52. **(B)** The domain organization and conserved protein domain of the ERF genes in the subclade including SlERF52. The two conserved sequences within the AP2/ERF domain (shown in red and light blue boxes) are shown in the lower panel. Red letters indicate conserved amino acid residues present in more than 50% of the aligned sequences. **(C)** The logo of two sequence motifs is shown by red and light blue boxes shown in panel **(B)**. The height of the superposition reflects the conservatism of the sequence, and the height of each marker reflects the frequency that amino acid residue appears.

Ethylene response factors are unique transcriptional regulators with various functions in plants and are typically encoded by members of a multigene family. According to the classifications for Arabidopsis ERFs, the family members have been divided into ERF and DREB, which together comprise ten distinct groups (designated from I to X) (Sakuma et al., 2002; Nakano et al., 2006). To clarify the phylogenetic relationships between the genes in both tomato and *C. campestris* ERF families, we performed multiple alignment analyses using amino acid sequences of the ERF family members and constructed a phylogenetic tree based on the alignment (Figure 3A). In this analysis, two *C. campestris* members (Cc003190 and Cc027208, hereinafter called CcERF52A and CcERF52B, respectively) branched into a single clade, which included SlERF52 in DREB group II. Interestingly, the alignment indicated that the AP2/ERF domains of CcERF52A possessed sparse homology to the consensus sequence (Figures 3B,C).

### Expression of CcERF52A and CcERF52B in Development of Haustorium

**FIGURE 4:**
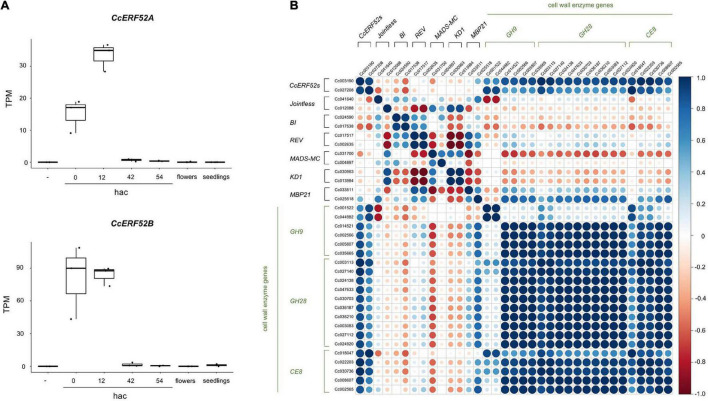
Characterization of *CcERF52A* and *CcERF52B* expression. **(A)** Expression patterns of the *C. campestris SlERF52* orthologous genes during the haustorial invasion. The abundance of the transcripts in each stage of haustorial invasion, flower bud clusters (shown as flowers), and seedlings were measured by mapping RNA-Seq reads and expressed as TPM. “-” indicates the epidermal and cortical cells of *C. campestris* without contact with the host plant. **(B)** Correlation analysis of the *C. campestris* orthologous regulator and cell wall enzyme genes, which are implicated in abscission of tomato, in different stages of haustorium development. Pearson correlation analysis was conducted using the Corrplot package in R. GH9, GH28, and CE include endo-β-1,4-glucanases, polygalacturonases and pectin methylesterases, respectively.

*CcERF52A* and *CcERF52B* were predominantly expressed at the initial invasion stage, during which haustorium penetrated the host tissue (Figure 4A). The expression patterns of *CcERF52A* and *CcERF52B* were consistent with those of the cell wall enzyme genes encoding endo-β-1,4-glucanases, polygalacturonases and pectin methylesterases (Figure 4B). Interestingly, the expression of *CcERF52A* and *CcERF52B* was not detected in the flower bud cluster (Figure 4A), indicating that these orthologs specifically function as transcriptional regulators of haustorial invasion. Additionally, our previous transcriptome analysis which used *in vitro* system indicated that interaction of *Cuscuta* with host tissues enhanced the expression levels of *CcERF52A* and *CcERF52B* during the invasion stage (Supplementary Figure 1; Kaga et al., 2020). We also selected the tomato abscission-associated regulators based on a model originally presented by Ito and Nakano (2015), and identified some *C. campestris* orthologs of these regulators, such as *Jointless, BI, REV* (Supplementary Table 6). However, no significant correlation was observed between the expression patterns of these regulators and cell wall genes encoding enzymes during haustorial invasion (Figure 4B).

## Discussion

The acquisition of haustoria is a critical evolutionary process for the establishment of plant parasitism. Sun et al. (2018) showed that many orthologous genes that are principally expressed in *Cuscuta* haustorium are also principally expressed in the roots of phylogenetically related autotrophic plant species. This suggests that *Cuscuta* haustorium evolution may have occurred by changing the expression mode of genes involved in root development. Similarly, comparative transcriptome analyses of root parasitic plants revealed that parasitism genes, which were defined as having enhanced expression in haustoria, are primarily derived from root tissue (Yang et al., 2015). The root is a highly specialized organ for the uptake and transfer of water and solutes, and is, therefore, a useful source for the evolutionary acquisition of haustorial function as a feeding organ. In the root parasitic plants, haustoria are generated from the apex of the primary root or lateral root extensions, supporting the idea that the haustorial structure evolved through changes in root development (Yoshida et al., 2016).

On the other hand, haustoria have evolved various sophisticated traits for parasitism. For example, haustoria develop into search hyphae that elongate *via* tip growth within the host tissue. The elongation of search hyphae is like the intrusive growth of pollen tubes in the flowers (Vaughn, 2003; Yang et al., 2015). This similarity is supported by the haustorial expression of *C. campestris* orthologous genes, which are expressed in the flowers of related non-parasitic plants (Yang et al., 2015; Sun et al., 2018).

**FIGURE 5:**
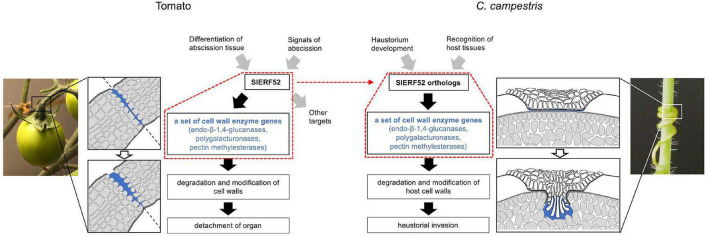
Model showing SlERF52-dependent regulatory module for the degradation and modification of cell walls in tomato abscission and *C. campestris* haustorial invasion. Red dot frames indicate SlERF52-dependent regulatory module. Transcriptional regulation in tomatoes is based on a model originally presented by Ito and Nakano (2015).

Haustorial invasion is also facilitated by mechanical action and by biochemical degradation and modification of host cell walls (Nagar et al., 1984; Olsen et al., 2016; Hozumi et al., 2017). Many genes encoding cell wall degrading and modifying enzymes were upregulated haustorium (Ranjan et al., 2014; Ikeue et al., 2015; Jhu et al., 2022). Furthermore, the dynamics of cell wall components were observed by the penetration of haustorium into the host tissue (Johnsen et al., 2015; Hozumi et al., 2017). Previously, we identified the cell wall enzyme genes principally expressed during the haustorial invasion of *C. campestris* and suggested that the orthogroups of these cell wall enzyme genes have been implicated in the abscission of closely autotrophic plants (Yokoyama et al., 2021). Because plant cell wall enzymes work collaboratively as a unique group for each biological event (Yokoyama, 2020), the haustoria of *Cuscuta* species may have recruited a unique set of cell wall enzyme genes that are involved in abscission. Identification of the *C. campestris* orthologous *SlERF52s* supports that the evolutionary acquisition of cell wall enzymatic activities in *Cuscuta haustoria* is related to changes in the transcriptional regulation of abscission processes since SlERF52 regulates the expression of a set of abscission-associated cell wall enzyme genes in tomato (Figure 5). More interestingly, the expression levels of *CcERF52A* and *CcERF52B* were significantly enhanced under the presence of the host tissues. We previously demonstrated that the host-produced ethylene is required for the proper growth of search hyphae in *C. haustoria* (Narukawa et al., 2021). In tomatoes, the expression levels of *SlERF52* are unlikely to be affected by ethylene production during abscission (Nakano et al., 2014). In *C. haustoria*, however, transcriptional regulation of *CcERF52A* and *CcERF52B* might be directly or indirectly linked to the regulatory network involving the ethylene pathway for elongation of search hyphae. Host recognition by haustoria is hypothesized to trigger the activation of multiple cascades and facilitate effective penetration into the host tissue.

We found other *C. campestris* orthologous regulators, including the earlier regulators that regulate abscission in tomatoes. However, we did not find a significant positive expression correlation between these orthologous regulators and cell wall enzyme genes during haustorium development. This result suggests that the haustorium of *Cuscuta* evolutionarily recruited only specific SlERF52-dependent regulatory mechanisms for the degradation and modification of cell walls (Figure 5). In tomatoes, it was successfully demonstrated that suppression of *SlERF52* decreased the transcript levels of particular cell wall enzyme genes (Nakano et al., 2014). Further in-depth investigations, including loss-of-function mutation, will be required to similarly show that CcERF52A and CcERF52B target the orthogroup of the cell wall enzyme genes in *C. campestris*. Since stable gene knockout approaches for *Cuscuta* plants is currently not available, it has not been possible to access *in vivo* function of CcERF52A and CcERF52B. However, it has recently been reported that the host-derived siRNAs could successfully down-regulate target gene transcription in *C. campestris, via* a host-induced gene silencing (HIGS) system (Jhu et al., 2022). One of the challenges is the use of HIGS system for knockdown of CcERF52A and CcERF52B in *C. campestri*s. Further investigation of the relationship between CcERF52s and the cell wall enzyme genes will be needed to support our hypothesis.

Abscission processes are governed not only by cell wall enzyme activities to ensure cell separation, but also by multiple cell differentiation processes for the detachment of an organ (Maity et al., 2021). For example, a protective layer involved in the thickening and lignification of cell walls is formed to protect the newly exposed surface during abscission (Lee et al., 2018). Since multiple functions of abscission may impede the progress of haustorium development, *Cuscuta* plants inevitably select only SlERF52-dependent regulatory mechanisms for the degradation and modification of cell walls. Furthermore, in tomatoes, SlERF52 is most likely to target several genes other than cell wall enzyme genes (Ito and Nakano, 2015; Wang et al., 2021). In this respect, the high rate of non-synonymous substitution of conserved residues in CcERF52A may be associated with the evolution of specialized regulatory functions to express cell-wall enzyme genes.

## Conclusion

The haustorium of *Cuscuta* is thought to have evolved from root tissues, based on the presence of large proportions of genes that are normally involved in root development. However, some traits of *Cuscuta* haustorium are markedly different from those of its root system. Haustorium may have evolved various parasitic traits through the recruitment of functional modules. Our findings provide valuable information for further studies exploring and understanding of haustorium evolution.

## Data Availability Statement

The datasets presented in this study can be found in online repositories. The names of the repository/repositories and accession number(s) can be found below: https://www.ddbj.nig.ac.jp/, DRR353312–DRR353329.

## Author Contributions

RY, TK, KA, and KN designed the research and wrote the manuscript. RY, TY, JP, and KA performed the research. RY, TK, JP, and KA analyzed the data. All authors discussed the results, reviewed the article, and approved the final article.

## Conflict of Interest

The authors declare that the research was conducted in the absence of any commercial or financial relationships that could be construed as a potential conflict of interest.

## Publisher’s Note

All claims expressed in this article are solely those of the authors and do not necessarily represent those of their affiliated organizations, or those of the publisher, the editors and the reviewers. Any product that may be evaluated in this article, or claim that may be made by its manufacturer, is not guaranteed or endorsed by the publisher.

## Footnotes

1
https://www.bioinformatics.babraham.ac.uk/projects/fastqc/

2
https://www.arabidopsis.org/tools/go_term_enrichment.jsp

3
https://trace.ddbj.nig.ac.jp/dra/index_e.html/DRA009453

4
https://www.plabipd.de

5
http://planttfdb.gao-lab.org

6
https://itol.embl.de

7
http://meme.nbcr.net/meme/

## Abbreviations

TF: transcription factor
DREB: dehydration response element-binding factors
DEG: differentially expressed genes
TPM: transcripts per million
LMD: laser capture microdissection.
