# Supplementary figures and images for: Regulatory Modules Involved in the Degradation and Modification of Host Cell Walls During Cuscuta campestris Invasion
Source: Front Plant Sci. 2022 Jul 6;13:904313. doi: 10.3389/fpls.2022.904313 (PMC9298654; doi:10.3389/fpls.2022.904313)

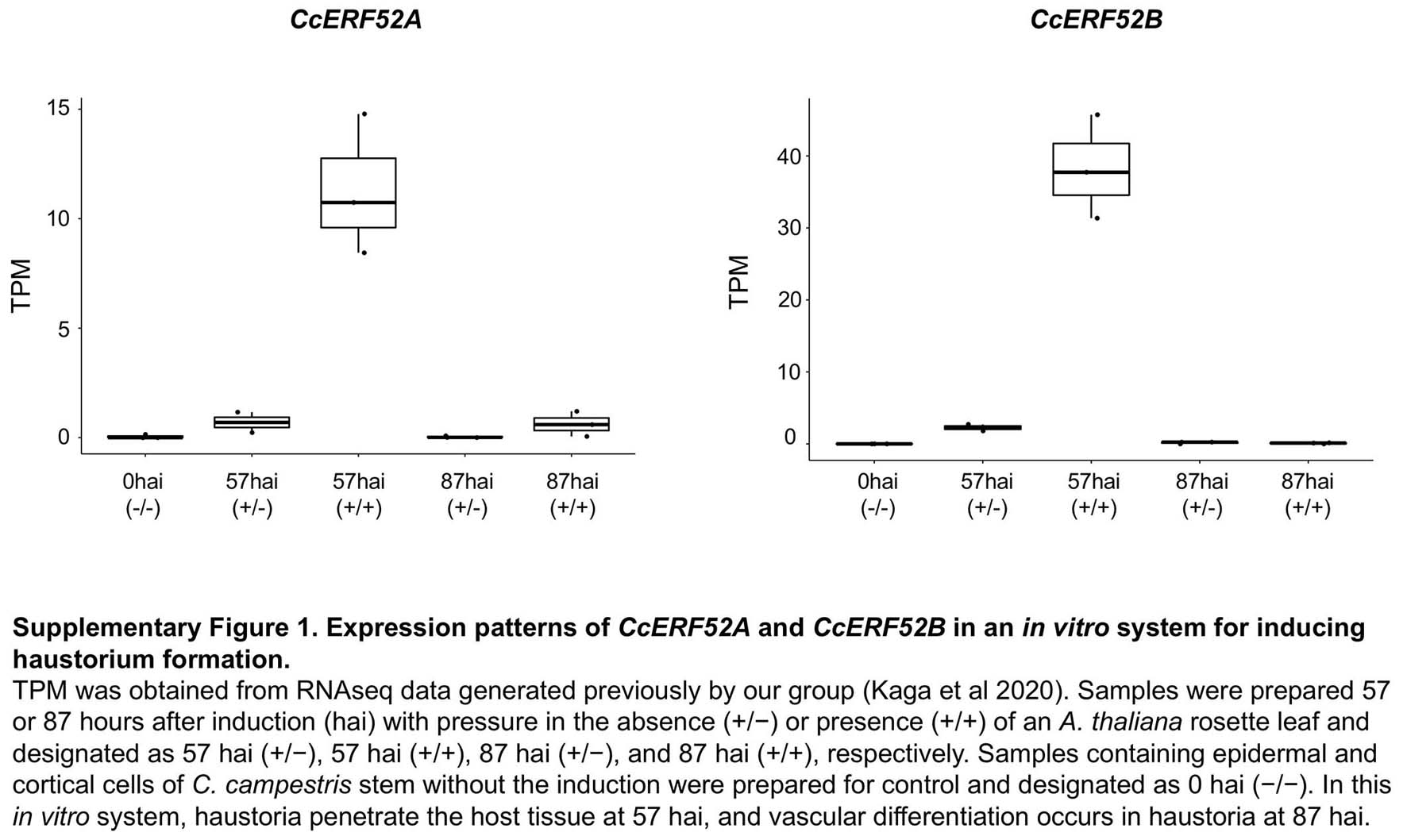

Supplement: Supplementary file 1 [file Image_1.JPEG]
